# Study of Lithium-Extraction Systems Based on Benzo-15-Crown-5 Ether and Alkylimidazolium-Based Ionic Liquid

**DOI:** 10.3390/molecules28030935

**Published:** 2023-01-17

**Authors:** Alexey A. Bezdomnikov, Liudmila I. Demina, Lyudmila G. Kuz’mina, Galina V. Kostikova, Valeriy I. Zhilov, Aslan Yu. Tsivadze

**Affiliations:** 1 Frumkin Institute of Physical Chemistry and Electrochemistry of Russian Academy of Sciences, Leninsky Pr. 31-4, Moscow 119071, Russia; 2 Kurnakov Institute of General and Inorganic Chemistry of Russian Academy of Sciences, Leninsky Pr. 31, Moscow 119991, Russia

**Keywords:** lithium extraction, lithium isotopes, benzo-15-crown-5 ether, complex synthesis, FTIR spectroscopy

## Abstract

The extraction of lithium from aqueous solutions of LiNTf_2_ and LiCl salts using benzo-15-crown-5 ether (B15C5) as an extractant in [C8mim][NTf_2_] ionic liquid was studied. The transition of the extractant into the aqueous phase and the distribution of Cl^−^ ions during lithium extraction from LiCl solutions were determined. LiNTf_2_ complexes with B15C5 with different LiNTf_2_:B15C5 ratios were isolated for the first time and characterized via X-ray diffraction and IR spectroscopy. Differences in the extraction process of LiCl and LiNTf_2_ were determined via an infrared spectroscopic study of the extraction systems.

## 1. Introduction

Currently, an active search for systems to produce pure lithium-7, which is widely used in the nuclear power industry, is underway. The most promising method of lithium-7 production is considered to be liquid extraction by crown ethers [1,2,3,4,5,6,7,8].

As was reported in [9,10,11], the use of different lithium salts affects the extraction efficiency, isotope separation, and crown ether distribution between phases. Analysis of a number of lithium salts (LiCl, LiBr, LiI, LiClO_4_, and LiSCN) in systems with benzo-15-crown-5 (B15C5)–chloroforms (CHCl_3_) [10] showed that as the anion radius and its lipophilicity increase, there is an increase in the distribution coefficient of lithium. Significant transition of B15C5 leads to a decrease in the efficiency of lithium isotope separation in extraction processes [9,10].

The use of crown ethers in combination with ionic liquids is a promising alternative [12,13,14,15,16,17] to the volatile and toxic solvents, such as CHCl_3_, commonly used in extraction processes. In [18], a large number of ionic liquids were considered as co-extractants with dibenzo-15-crown-5 ether (DB15C5), and the highest lithium distribution ratios were observed with [C4mim][NTf_2_]. The same investigation reported a linear correlation between the lithium content in the organic phase and the transfer of [C4mim]^+^ into the aqueous phase due to ion exchange, which leads to ionic liquid losses.

When lithium salts with different anions (CF_3_COOLi, LiNO_3_, LiBr, LiI, LiCl, LiClO_4_, and LiNTf_2_) extracts by the DB15C5–[C6mim][NTf_2_]-anisol mixture [11], the different extractabilities of lithium are observed; however, the different extractability of lithium is observed, however the difference in isotope effects negligible. The maximum distribution coefficients of lithium are characteristic of the system with LiNTf_2_. There are inconsistent data in the literature regarding the composition of extracted complexes with lithium and crow-ethers in the presence of ionic liquids [2,11,18,19].

In this work, the differences in extraction of LiCl and LiNTf_2_ by B15C5 solution in [C8mim][NTf_2_] were revealed. Solid complex compounds of LiNTf_2_ with B15C5 with different LiNTf_2_:B15C5 ratios, which can potentially be formed during extraction in ionic liquids with NTf_2_^−^ anions, were obtained. The obtained complexes were investigated and characterized using the X-ray diffraction method and FTIR ATR spectroscopy. FTIR spectra analysis of the extraction systems was performed to determine the composition of the extracted complexes.

## 2. Results and Discussion

### 2.1. Extraction

**Figure 1:**
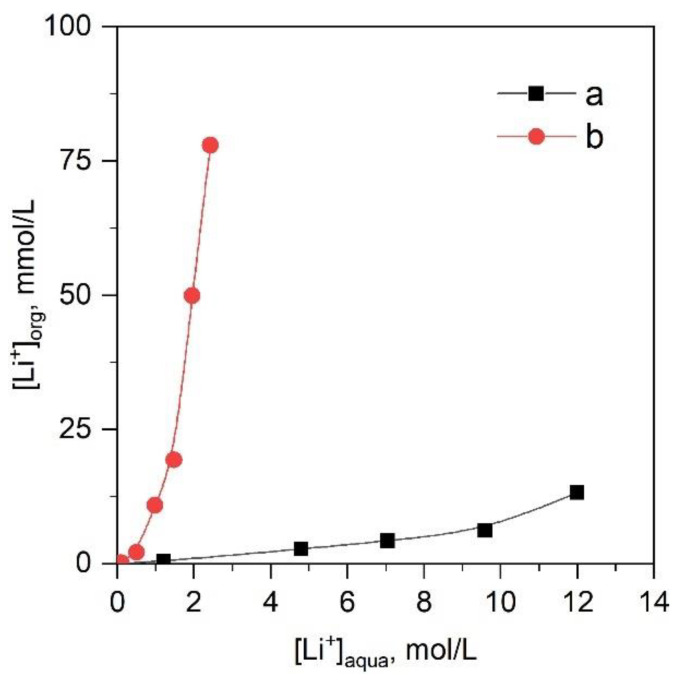
Blank extraction of lithium in systems: (**a**) LiCl-[C8mim][NTf_2_], (**b**) LiNTf_2_-[C8mim][NTf_2_]. O/A = 1, T = 23 ± 2 °C.

According to the literature data, the blank extraction of lithium chloride by chloroform [20] does not exceed 0.3 mg/L; i.e., it is negligible. We estimated the blank extraction of LiCl and LiNTf_2_ by ionic liquid [C8mim][NTf_2_] (Figure 1). The extraction of LiCl with the ionic liquid was much higher than with chloroform and achieved 14 mmol/L (98 mg/L). In the case of LiNTf_2_, the extraction was even greater and achieved 79 mmol/L (0.55 g/L). The difference between LiCl and LiNTf_2_ blank extraction is associated with the higher lipophilicity of LiNTf_2_. Such high blank extraction of LiNTf_2_ by ionic liquid at high salt concentrations in the equilibrium aqueous phase can critically affect the lithium isotope separation, contributing to lithium extraction but reducing the efficiency of isotope separation by crown ether. Therefore, in case of LiNTf_2_, it is necessary to avoid high salt concentrations in the equilibrium aqueous phase and operate in concentration intervals up to 2 mol/L.

**Figure 2:**
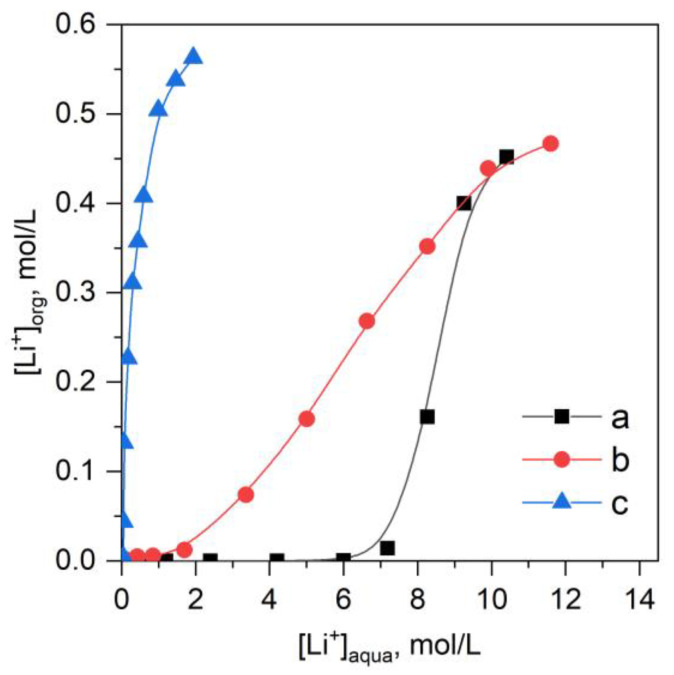
Lithium extraction isotherms in the systems: (**a**) LiCl-B15C5-CHCl_3_, (**b**) LiCl-B15C5-[C8mim][NTf_2_], (**c**) LiNTf_2_-B15C5-[C8mim][NTf_2_]. C^0^_B15C5_ = 0.5 mol/L, O/A = 1, T = 23 ± 2 °C.

Extraction of lithium chloride by B15C5 when using chloroform as an organic solvent was observed only at LiCl concentrations higher than 8 mol/L (Figure 2); extraction isotherm of LiCl had a sharp S-shape for different B15C5 concentrations [20]. Replacing chloroform as the organic solvent with an ionic liquid [C8mim][NTf_2_] in the extraction of LiCl by B15C5 (0.5 mol/L) solution substantially increased lithium extraction in the initial isotherm region (Figure 2). When extracting LiNTf_2_ (Figure 2), the extraction isotherm shape changed, and high values of the lithium distribution coefficient were observed in the initial isotherm region.

Extractant transition to the aqueous phase is extremely important when considering extraction [21]. As was described earlier in [9], B15C5 transition into the aqueous phase during LiCl extraction, when CHCl_3_ is used as a solvent, reaches its maximum at a LiCl concentration of 6.5–8 M, when it reaches 20% of the initial concentration of crown ether. As was shown in [10], an increase in the initial concentration of crown ether in the organic phase increases the relative transition of the extractant to the aqueous phase at an initial B15C5 concentration of 0.78 mol/L in chloroform for the system with LiSCN, 12% of the extractant transits, and at its initial B15C5 content of 1.5 mol/L-33%.

**Figure 3:**
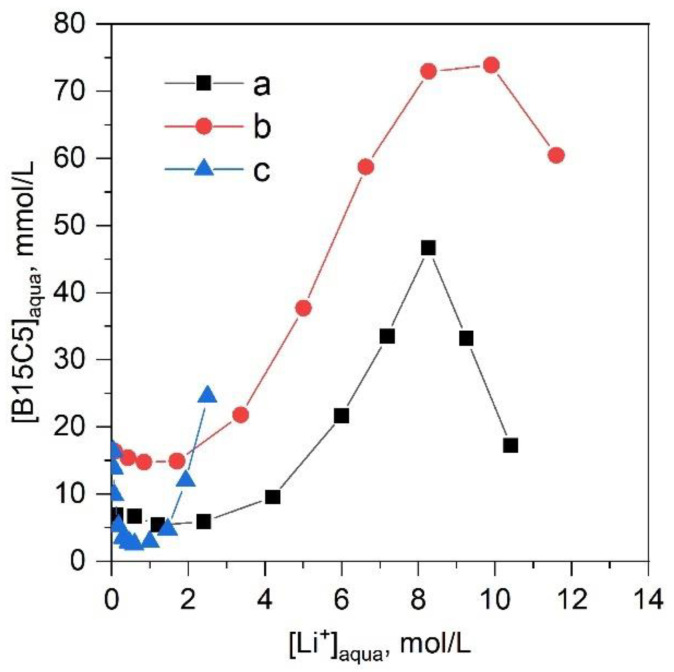
Transition of extractant to the aqueous phase in extraction systems; (**a**) LiCl-B15C5-CHCl_3_, (**b**) LiCl-B15C5-[C8mim][NTf_2_], (**c**) LiNTf_2_-B15C5-[C8mim][NTf_2_]. C^0^_B15C5_ = 0.5 mol/L, O/A = 1, T = 23 ± 2 °C.

The transition of B15C5 (C^0^_B15C5_ 0.5 mol/L) into the aqueous phase was determined for the LiCl-B15C5-CHCl_3_ extraction system (Figure 3). The obtained dependence was similar to that previously described in the literature; the maximum transition of the crown ether to the aqueous phase under studied conditions was 9.4%. Replacing CHCl_3_ with [C8mim][NTf_2_] substantially increased the extractant transition to the aqueous phase over the entire range of LiCl concentrations, with the maximum transition being 14.8%. The system with LiNTf_2_ showed a U-shaped dependence of the extractant transition to the aqueous phase on the concentration of lithium in the aqueous phase, with a local minimum of 0.5%. Further increase in lithium concentration lead to a strong increase in the concentration of B15C5 in the aqueous phase, which exceeded the transition shown in systems with LiCl. When using solutions with LiNTf_2_ concentrations of more than 2 mol/L, poor separation of the extraction system and phase inversion was observed. 

**Figure 4:**
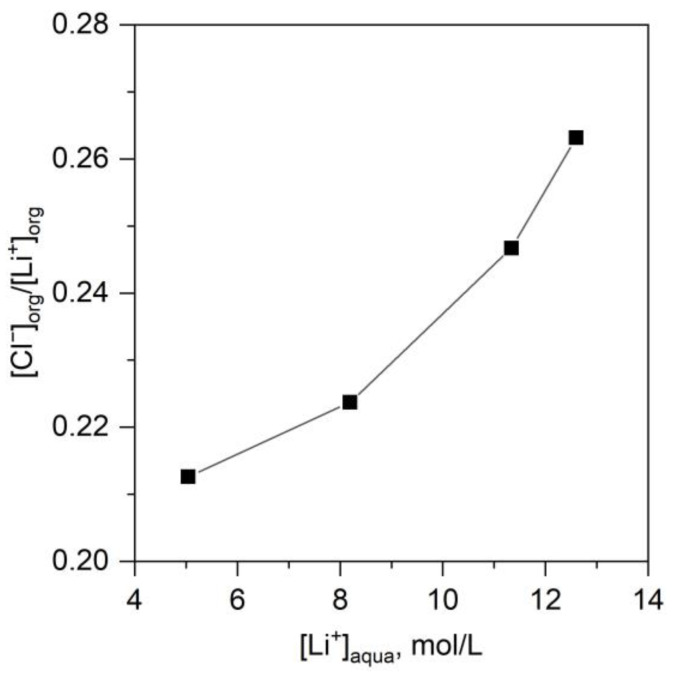
Ratio of equilibrium concentrations of chloride anions to lithium cations in the organic phase as a function of lithium concentration in the equilibrium aqueous phase. Extraction system LiCl-B15C5-[C8mim][NTf_2_], C^0^_B15C5_ = 0.5 mol/L, O/A = 1, T = 23 ± 2 °C.

Since in the extraction system during LiCl extraction two anions (Cl^−^ and NTf_2_^−^) are presented, to understand the extraction process it is important to determine the distribution of anions between phases. The ratio of equilibrium concentrations of chloride anions and lithium cations in organic phases were determined for the extraction system LiCl-B15C5-[C8mim][NTf_2_] with the help of a mass-spectroscopy (Figure 4). The relative content of chloride anion in the extract practically did not change and was never more than 28% of the concentration of lithium cations. This indicated ion exchange between the ionic liquid and lithium chloride, with a [C8mim]Cl compound being formed. [C8mim]Cl preferentially transited into the aqueous phase. Lithium predominantly formed a complex compound, with NTf_2_^−^ as the counterion.

Therefore, the system with LiNTf_2_ has significant advantages over the systems with LiCl, provided that the concentration of LiNTf_2_ does not exceed 2 M. A further increase in concentration lead to a number of negative effects: (1) a sharp increase in blank extraction (which can lead to a decrease in extraction selectivity); (2) a sharp increase in the transition of the extractant into the aqueous phase; and (3) poorer phase separation and phase inversion.

### 2.2. X-ray Diffractometry

**Figure 5:**
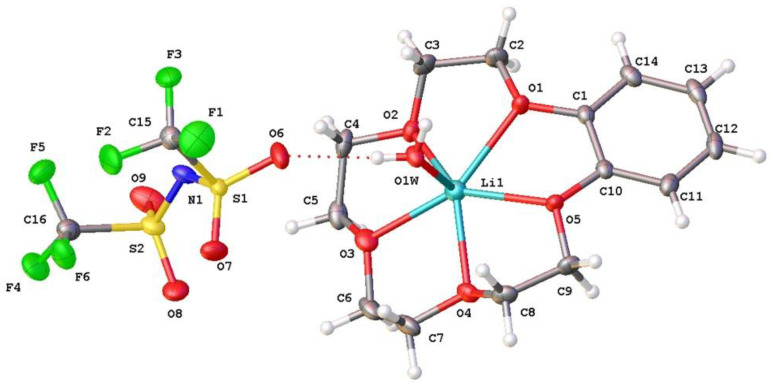
Structure of formula units in crystal (***I***). Ellipsoids of l.s. temperature atomic displacements are shown at 50% probability level.

Solid complex compounds between LiNTf_2_ and B15C5 potentially forming on the extraction in ionic liquids with NTf_2_^−^ anions at different LiNTf_2_:B15C5 ratios were selected. The X-ray structure of that with the ratio 1:1 (***I***) is shown in Figure 5. 

The crystal consisted of NTf_2_^−^ anions and lithium cations coordinated by five oxygen atoms of the B15C5 and the water molecule in the apical position of the pentagonal pyramide. The lithium atom was displaced from the basis plane by 0.28 Å; the displacements of the crown ether oxygen atoms from their mean plane varied within the range of 0.06–0.19 Å. In the coordination polyhedron, the Li…O1w with the water oxygen was the shortest one (Li…O1w 1.919(2) Å). The distances between the crown ether oxygen atoms were much longer. The Li…O distances with the O1 and O5 atoms in the sp^2^ hybridization state (angles C-O-C 117.14(9) and 117.39(9)°) were longer (2.278 and 2.269 Å) than those with the O2, O3, and O4 atoms in the sp^3^ hybridization state, which were equal to 2.197(2), 2.206(2), and 2.218(2) Å, respectively. The angles at the O2, O3, and O4 atoms varied within the range of 112.7–114.1°. Since the atom in the sp^3^ hybridization state had a bigger covalent radius than that of the atom in the sp^2^ hybridization state, one should expect an opposite Li…O(sp^3^) to Li…O(sp^2^) ratio. This discrepancy was apparently due to the higher conformational rigidity of the fragment of the macrocycle involving the O1 and O5 atoms, the last ones being involved in a conjugation with the benzene ring. A similar peculiarity was also observed in the earlier-studied structures of lithium with B15C5 and water in the apical position of lithium coordination polyhedron and various counterions (Cl^−^, I^−^, ClO_4_^−^, NO_3_^−^, BF_4_^−^) [22]. In this study, a rather complicated NTf_2_^−^ anion was used. In contrast to simple inorganic anions, anion A is therefore able to coordinate the metal atom to compete with B15C5 for lithium coordination. 

This anion may coordinate with lithium in several ways. One of these is bridging the function of the ligand through oxygens of different SO_2_ fragments: Li_1_…[O_1_=S(O)CF_3_-N-(CF_3_(O)S=O_2_]…Li_2_. One other way is using a chelate coordination, when the same oxygen atoms are coordinated with the same lithium atom Li_1_…[O_1_=S(O)CF_3_-N-(CF_3_(O)S=O_2_]…Li_1_ [23,24,25,26]. There have also been known cases of the ligand monodentate coordination [23]. However, cases demonstrating the mixed chelate-bridging function of the NTf_2_^−^ anion in lithium complexes are more widespread [27,28,29]. Cases of separated cations and anions are also available [30]. Lastly, the theoretical variant of an interaction of the cation with the anion nitrogen atom bearing a negative charge should also be taken into account. The structures of these types are not available for lithium in the Cambridge Structural Database. However, in principle, such a possibility cannot be excluded, and this coordination was actually observed for Cs [31]. 

**Figure 6:**
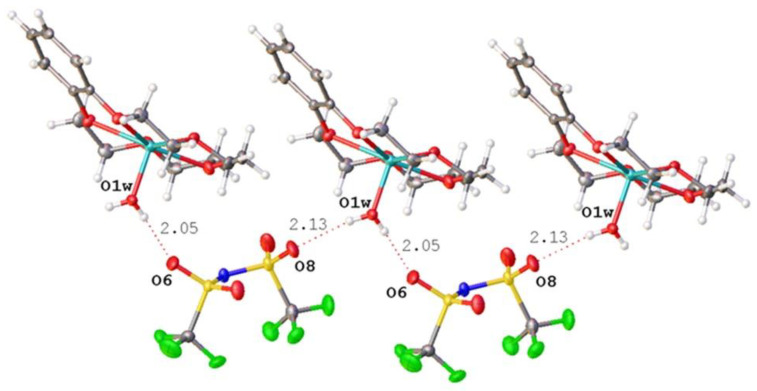
Chain formed by cations and anions due to hydrogen bonds O-H…O.

In structure (***I)*,** there were no direct interactions between the lithium atoms and the anions, but cations and anions were linked in infinite chains through hydrogen bonds O-H…O between coordinated water molecules and SO_2_ fragments of the anions (Figure 6).

**Figure 7:**
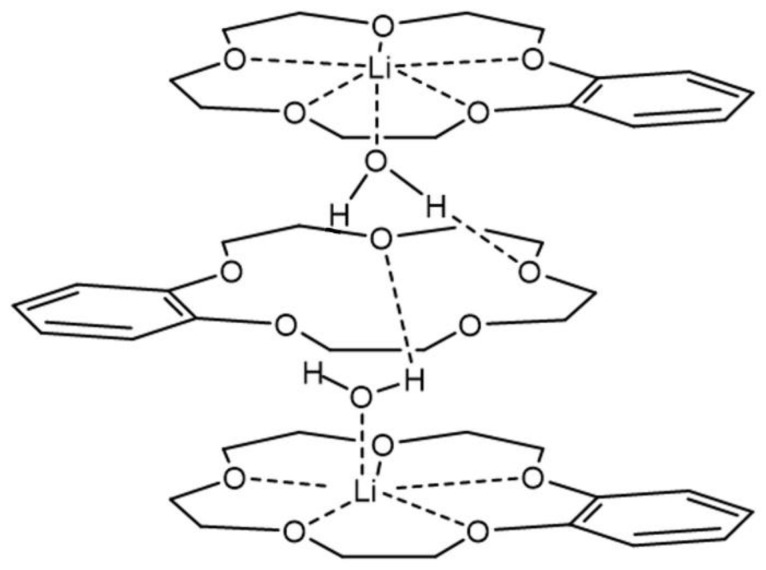
Scheme of the three-decked cation in (***II_mon_***) and (***II_tr_***).

Compound (***II***) was obtained in two crystal modifications: (***II_mon_***) and (***II_tr_***). The cation represented a complicated three-decked species, each layer of which involves a B15C5 fragment, and the layers were linked via coordinated water molecules (see the scheme in Figure 7).

**Figure 8:**
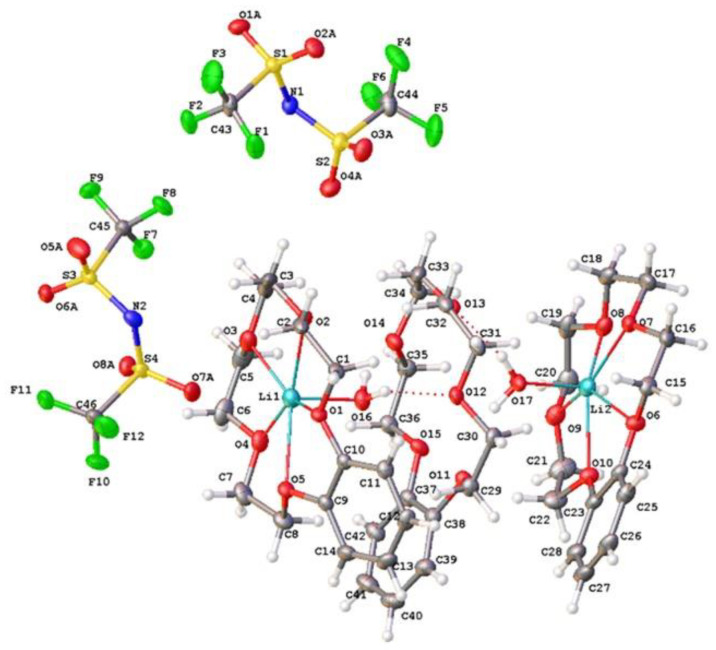
Structure of formula units of crystal (***II_mon_***). Ellipsoids of atomic l.s. thermal displacements are given at the 50% probability level.

**Figure 9:**
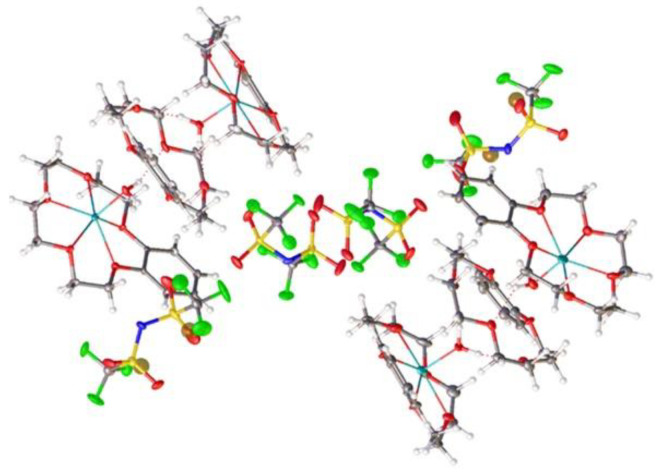
Structure of components of crystal unit cell in (***II_tr_***). Ellipsoids of atomic l.s. thermal displacements are given at the 50% probability level.

Figure 8 and Figure 9 show the components of the crystal unit cell in crystals (***II_mon_***) and (***II_tr_***), respectively. All three decks were formed by B15C5. Two outer decks contained lithium cations coordinated by crown ether and water molecules. Each water molecule occupied an apical position of the lithium polyhedron and was oriented towards the central deck to form hydrogen bonds O-H…O with its crown ether oxygen atoms. The “upper” and “lower” halves of the cation were not identical; the water molecule of the “upper” half formed two hydrogen bonds O(H_2_O)-H…O(crown) with the central crown ether, whereas the water molecule of the “lower” half formed only one hydrogen bond. Monoclinic modification contained one independent formula unit (one cation and one anion), whereas triclinic modification contained two formula units with very close corresponding geometrical parameters. In the cation of the monoclinic modification, the H…O distances were equal to 1.95, 2.01, and 2.20 Å. A similar situation was observed in the triclinic modification: the corresponding H…O distances were 2.10, 2.02, and 2.07 Å in one independent molecule, and 2.09, 2.01 and 2.02 Å in another one. In the monoclinic modification (***II_mon_***), the opposite Li-O(sp^2^) to Li-O(sp^3^) ratio observed in structure (***I***) was less pronounced. The first-type distances were equal to 2.226(3), 2.414(4), 2.253(4), and 2.357(4), whereas the second-type distances were 2.310(4), 2.124(4), 2.145(4), 2.247(4), 2.151(4), and 2.224(4) Å. In the triclinic modification (***II_tr_)*,** neither of the type’s distances were widely scattered. The Li…O distances with water molecules in the apical positions were equal to 1.912(9), 1.929(8), 1.924(8), and 1.929(8) Å. 

**Figure 10:**
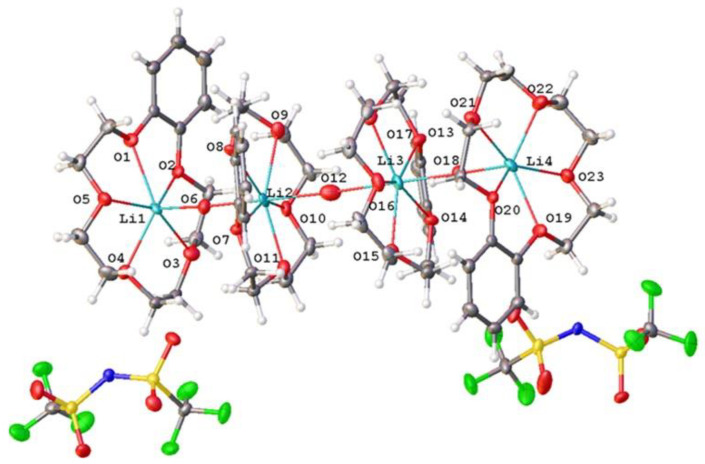
Structure of cation and anions of (***III***). Ellipsoids of atomic l.s. thermal displacements are given at the 50% probability level.

The structure of (***III***) was more complicated. The independent part of the crystal unit cell consisted of two anions and a four-decked cation (Figure 10), with the last ion being two-charged. Since in each of them a central peak corresponding to a lithium atom was located and the total charge of the cation as equal to two, one should conclude that the corresponding Li position had partial (~0.5) occupation. This means that two lithium atoms were distributed over four positions. The coordination of terminal atoms Li1 and Li4 was supplemented to the pentagonal pyramidal due to the water oxygen atoms O6 and O18. The central Li2 and Li3 atoms had pentagonal bipyramidal coordination. The axial position occupied the oxygen atoms O6, O12, and O18 of the water molecules. The distances Li1…O6 and Li4…O18 (1.899(8) and 1.953(8) Å, respectively) were the shortest of the Li…O distances. The distances of Li2…O6, Li2…O12, Li3…O12, and Li3…O18 were equal to 2.106(8), 2.294(8), 2.325(9), and 2.117(9) Å, respectively, with the longest one being with the central O12 atom. These distances were determined with a high error because very light Li atoms have half occupation. Additionally, it is possible that the four lithium atoms had somewhat different occupations, although their total occupation was equal four. This suggestion is indicated by the fact that the thermal ellipsoid of O12 atom was greater than others and had an elongated shape along the O12…O18 line.

In compounds (***II***) and (***III),*** there was no direct interaction between cations and anions.

### 2.3. FTIR Spectroscopy of the Complexes **(I–III)**

As described above, X-ray diffraction showed isostructurality in the complex (***I***) with the majority of the lithium complexes with B15C5 reported in the literature [22,32]. This enables for the use of the FTIR spectrum of the previously isolated and characterized complex [Li(B15C5)(H2O)Cl] as a source of comparison in the analysis of the FTIR spectrum of complex (***I***).

**Figure 11:**
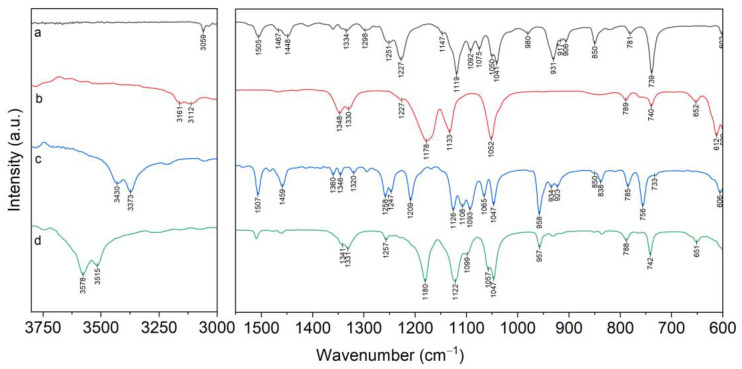
FTIR spectra: a—B15C5; b—[C8mim][NTf_2_]; c—[Li(B15C5)(H_2_O)Cl]; d—[Li(B15C5)(H_2_O)(NTf_2_)] (***I***).

Analysis of the structure of the complexes (***I***–***III***) using IR spectra has limitations because bands describing the vibrations of the donor atoms of B15C5 are overlapped by bands describing vibrations of the NTf_2_^−^ anion. This primarily concerns the regions ν(PhO): 1270–1230 cm^−1^ and ν_as_(COC): 1150–1040 cm^−1^. However, the comparison of the B15C5 and [C8mim][NTf_2_] spectra (Figure 11) allows for the detection of ranges from 1000 cm^−1^ to 800 cm^−1^ and from 3888 cm^−1^ to 3200 cm^−1^, where there are no overlapping bands.

The 1000–800 cm^−1^ range is the most significant for spectra analysis of B15C5 and its complexes as it is characterized by the presence of the combined stretching–deformation modes ρ(CH2) + ν(CO) + ν(CC) of the ethylene glycol fragments of the crown ether [33]. Furthermore, in this range of free B15C5 spectrum, the band ν_s_(COC) 981 cm^−1^ is located, which we used earlier as the most characteristic bands for describing the complexation [22,32,34]. This band shifts to the low-frequency region by 20–30 cm^−1^ in the spectra of lithium complexes with B15C5 with various anions (Cl^−^, I^−^, ClO_4_^−^, NO_3_^−^, BF_4_^−^, NCS^−^). The isolated position of this band makes it suitable for controlling the complexation process. The ν_s_(COC) band shift up to 957 cm^−1^ is also observed in spectrum of complex (***I***) (Figure 11). The spectrum of complex (***I***) in the range of 1000–800 cm^−1^ was similar to that of the earlier-described [Li(B15C5)(H_2_O)Cl], indicating that the conformations of B15C5 ligand on both complexes are similar. Indeed, X-ray diffraction data showed that the lithium cation in complex (***I***) formed coordination links with one molecule of B15K5 and one molecule of water. The Li…O(sp^3^) to Li…O(sp^2^) distance ratios and the position of the water molecule in the apical position of the pentagonal pyramid at the Li atom corresponded to those determined earlier for complexes with other anions. All five oxygen atoms of B15C5 in complex (***I***) participated in coordination. 

Different forms of the crystals (***II_mon_***) and (***II_tr_***) were detected during sample preparation for PCA. The crystals were mechanically separated under the microscope. The spectrometer and the ATR method made it possible to characterize the same selected crystal by both X-ray diffraction and FTIR. No differences in the IR spectra of (***II_mon_***) and (***II_tr_***) were detected. This was due to the fact that the complex cation in both (***II_mon_***) and (***II_tr_***) had the same structure, and the difference in the structure of compounds was due only to the spatial orientation of NTf_2_^−^ anions relative to the cation. The similarity between the IR spectra allowed us to combine these polymorphic forms under the single name of complex (***II***) when discussing the results. In complex (***II***), along with two terminal B15C5 molecules coordinated with lithium, there was a central crown ether molecule, which formed strong hydrogen bonds with coordinated water molecules. Complex (***III***) had the four-deck cation where central atoms Li1 and Li2 are pentagonal–pyramidal coordinated, while terminal ones Li3 and Li4 were pentagonal–bipyramidal coordinated.

Differences in the structure of the complexes (***I–III***) lead to significant changes in the IR spectra in the range of 1000–800 cm^−1^ (Table 1). Stretching mode ν_s_(COC) in the spectra of complexes (***II***) and (***III***) was observed as doublet bands at 963 and 954 cm^−1^ for complex (***II***), and at 965 and 947 cm^−1^ for complex (***III***), while in the spectra of complex (***I***) and B15C5, ν_s_(COC) appeared as singlet bands at 957 cm^−1^ and 981 cm^−1^, correspondingly. 

**Figure 12:**
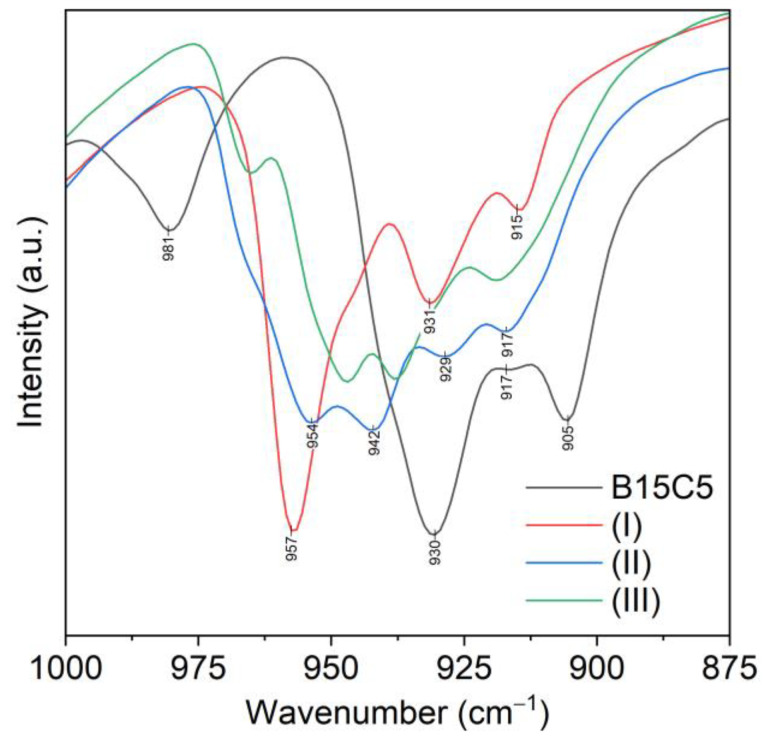
Fragments of FTIR spectra of B15C5; (***I***)—[Li(B15C5)(H_2_O)(NTf_2_)]; (***II***)—[Li(B15C5)_1.5_(H_2_O)](NTf_2_); (***III***)—[Li(B15C5)_2_(H_2_O)](NTf_2_).

The number of bands of the combined stretching–deformation modes in the range of 1000–800 cm^−1^ in spectra of complexes (***II–III***) was increased (Figure 12 and Figure S1), which can be explained by the presence of a greater variety of B15C5 conformation forms in (***II–III***) as compared to complex (***I***) and free B15C5. 

The position of the bands ν(OH) in the range of 3700–3000 cm^−1^ and δ(H_2_O) in the range of 1700–1600 cm^−1^ in spectra of all complexes (Figure S1) enables us to confirm the presence and binding strength of water molecules in the complexes.

Water molecules exhibit both electron donor and electron acceptor abilities in complexes (***I–III***). Water molecules exhibit electron donor properties, with the coordination bond formation of the oxygen atom of water with lithium in (***I–III***). The electron acceptor properties of water are realized when infinite chains of hydrogen bonds O-H…O are formed with the participation of water molecules and sulfonyl group of NTf_2_^−^ (***I***), or when coordinated water molecules bind with the hydrogen bonds of O-H…O coordination-free crown ether fragments (***II***). In the FTIR spectra of the complexes, this difference is illustrated by the different degrees of splitting and the position of the ν(OH) bands and the δ(H_2_O) band. The greater splitting of ν(OH) in spectrum of (***III***) demonstrates significant differences in distances from the terminal and central lithium atoms to the oxygen atom of coordinated water, which was also revealed earlier through the X-ray diffraction method (Table 2, Figure S1).

As shown by X-ray diffraction, the NTf_2_^−^ anion has different functions across complexes (***I–III***): in complex (***I***), the sulfonyl group of NTf_2_^−^ anion is bound to a coordinated water molecule, whereas in (***II***) and (***III***), it plays the role of a free anion. These differences are reflected in the position of the ν_as_(SO_2_) bands (Figure S1). In the spectra of complexes (***II***) and (***III***), the unbound sulfonyl group was described by two ν_as_(SO_2_) bands, which were located at 1356 cm^−1^ and 1336 cm^−1^ for (***II***), and at 1347 cm^−1^ and 1334 cm^−1^ for (***III***). These were also located in spectrum of ionic liquid [C8mim][NTf_2_] at 1347 cm^−1^ and 1330 cm^−1^, while the binding of one of the oxygen atoms of the sulfonic groups of the anion with a water molecule in complex (***I***) lead to a significant decrease in the band splitting of ν_as_(SO_2_) at 1331 cm^−1^ (shoulder 1341 cm^−1^). The ν_s_(SO_2_) band retained its 1180 cm^−1^ position in the spectra of (***I–III***). The results of the FTIR spectrum analysis of isolated complexes (***I–III***) were used to study lithium extraction.

### 2.4. FTIR Spectroscopy of Blank Extraction

**Figure 13:**
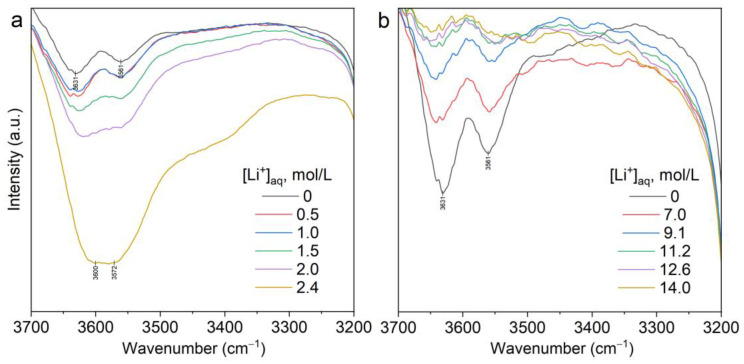
FTIR spectra of LiNTf_2_ (**a**) and LiCl (**b**) blank extraction in [C8mim][NTf_2_] with different [Li^+^]_aq._ contents.

The FTIR spectra of organic phases during blank extraction of LiNTf_2_ by solvent [C8mim][NTf_2_] are presented in Figure 13a and Figure S2. The presence of ν(OH) bands at 3630 cm^−1^ and 3560 cm^−1^ in the spectrum of the organic phase at [Li^+^]_org_ = 0 indicates that [C8mim][NTf_2_]*nH_2_O hydrates are formed when the ionic liquid is in contact with water. The increase in the concentration of lithium in the organic phase during blank extraction into the ionic liquid [C8mim][NTf_2_] from aqueous solutions of LiNTf_2_ was accompanied by an increase in the intensity of the ν(OH) bands. The increase in the intensity of these bands was accompanied by the appearance of a broad shoulder in the region of 3400 cm^−1^, which indicates the transition of water molecules in the organic phase as part of the aquated lithium ion [Li(H_2_O)_n_]^+^, which bonded with NTf_2_^−^. Therefore, it can be noted that the degree of blank extraction is correlated with the intensity of ν(OH) bands. 

Replacing NTf_2_^−^ anions with chloride anions in aqueous solutions of lithium salts leads to a significant decrease in blank extraction. Analysis of the FTIR spectra of the organic phases in this experiment showed that increasing the concentration of LiCl in the aqueous phase resulted in the dehydration of ionic liquid. As shown in Figure 13b and Figure S3, the intensity of the 3630 cm^−1^ and 3560 cm^−1^ bands decreases gradually, almost approaching zero at a lithium concentration of 14.0 mol/L in the aqueous phase. 

### 2.5. FTIR Spectroscopy of Extraction Systems with Crown Ethers

**Figure 14:**
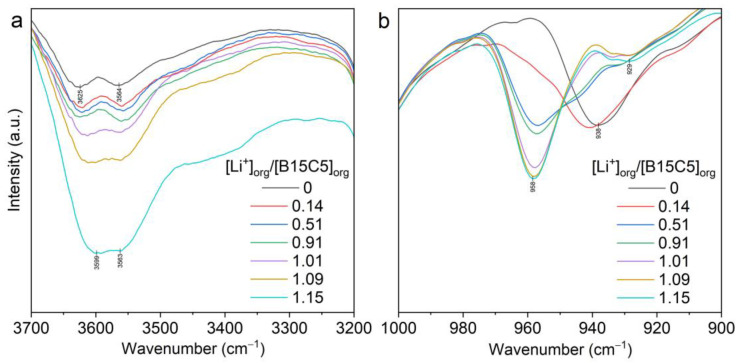
FTIR spectra of LiNTf_2_-B15C5 (0.5 mol/L)-[C8mim][NTf_2_] extract at the different [Li^+^]/[B15C5] ratios in the ranges of 3700–3200 cm^−1^ (**a**) and 1000–900 cm^−1^ (**b**).

The more informative range for the analysis of FTIR spectra of extracts in our investigations was 1000–880 cm^−1^ (Figure 14a and Figure S4).

It is possible to determine the structure of an extracted complex based on the analysis of combined stretching–deformation modes of ethylene glycol components of the crown ether, which were observed in this IR region and based on X-ray diffraction data for the earlier-isolated complexes (***I–III***). 

The medium-intensity band 938 cm^−1^ and weakly broadened bands 984 cm^−1^ and 913 cm^−1^ characterize free B15C5 in the spectrum of a 0.5mol/L solution of B15C5 in [C8mim][NTf_2_]. In the IR spectra of the extracts, the intensity of these bands decreased as the concentration of LiNTf_2_ in the aqueous phase progressively increased and the [Li+]_org_/[B15C5]_org_ in the organic phase increased (Figure 14b and Figure S4, respectively). Along with this, the appearance and increase in the intensity of bands 958 cm^−1^ and 930 cm^−1^ was observed. At [Li^+^]_org_/[B15C5]_org_ 0.91–1.01, the relative intensity of these bands stabilized, and the bands characterizing free B15C5 disappeared. This suggests the formation of a complex with a Li to B15C5 ratio of 1:1 in the organic phase. The observed increase in the intensity of ν(OH) bands in the range of 3700–3500 cm^−1^ (Figure 14a and Figure S4) is associated with the formation of the complex (***I***), which contains a water molecule. The appearance of the 3400 cm^−1^ shoulder is associated with a parallel blank extraction of [Li(H_2_O)_n_]^+^.

**Figure 15:**
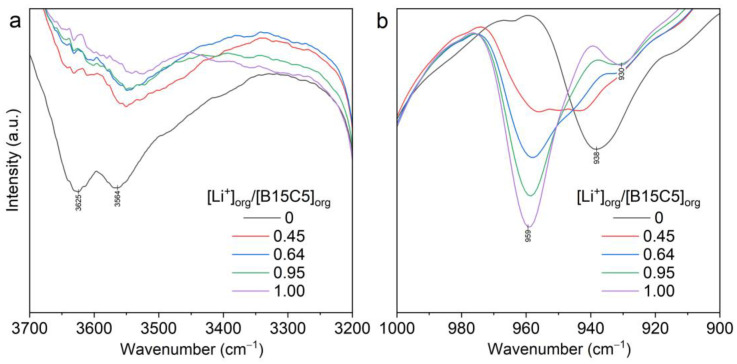
FTIR spectra of LiCl-B15C5 (0.5 mol/L)-[C8mim][NTf_2_] extracts at different [Li^+^]/[B15C5] ratios in the ranges of 3700–3200 cm^−1^ (**a**) and 1000–900 cm^−1^ (**b**).

A study of the extraction of lithium from LiCl aqueous solution using the same system was carried out. As the concentration of LiCl in the aqueous phase increased, the proportion of coordinated B15C5 in the organic phase increased. We observed similar behavior in the system with LiNTf_2._ The FTIR spectra revealed that as the intensity bands of coordinated B15C5 at 958 cm^−1^ and 930 cm^−1^ increased, and the intensity band of uncoordinated B15C5 at 913 cm^−1^, 938 cm^−1^, and 984 cm^−1^ (Figure 15b and Figure S5) decreased, the intensity of the ν(OH) bands in the range of 3700–3500 cm^−1^ decreased (Figure 15a and Figure S5), indicating the extraction of the complex in anhydrous form and the process of dehydration of the ionic liquid, which we noted in the study of the blank extraction from aqueous solution LiCl.

**Figure 16:**
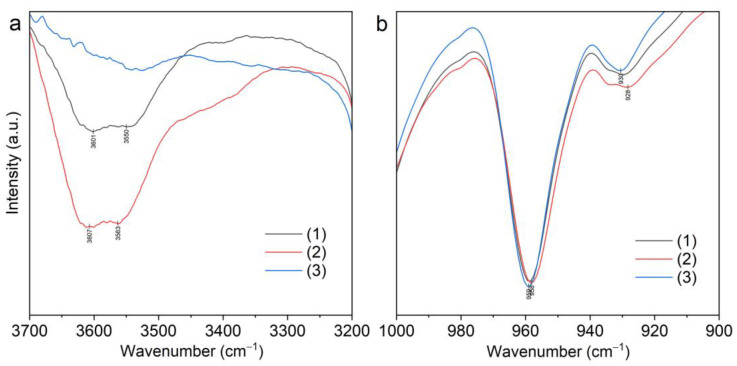
Fragments of FTIR spectra in the ranges of 3700–3200 cm^−1^ (**a**) and 1000–900 cm^−1^ (**b**): (1) solution of the complex (***I***) in [C8mim][NTf_2_] (0.5 mol/L); (2) extract of LiNTf_2_-B15C5 (0.5 mol/L)-[C8mim][NTf_2_]; (3) extract of LiCl-B15C5 (0.5 mol/L)-[C8mim][NTf_2_].

The identity of the spectra of extracts in both systems (with LiCl and LiNTf_2_) and the spectrum of 0.5 mol/L (***I***) in [C8mim][NTf_2_] (Figure 16b and Figure S6) in the range of 1000–800 cm^−1^ allows us to conclude that the B15C5 molecules in the lithium complexes formed in the extracts have the same conformation of ethylene glycol units as in complex (***I***).

The differences in the range of 3700–3400 cm^−1^ (Figure 16a and Figure S6) were caused by the presence of blank extraction of [Li(H_2_O)_n_]^+^ in investigated systems, the formation of [C8mim][NTf_2_]*nH_2_O solvates, and the absence of water molecules in the complex for the system with LiCl. 

In the process of extraction from LiCl aqueous solution, both complexes with Cl^−^ and with NTf_2_^−^ can be formed. However, we could not determine the anion of the extracted complex by spectroscopic analysis of the extracts. In the organic phase, [C8mim][NTf_2_] was used as the solvent, and Cl^−^ was not detected spectroscopically.

**Figure 17:**
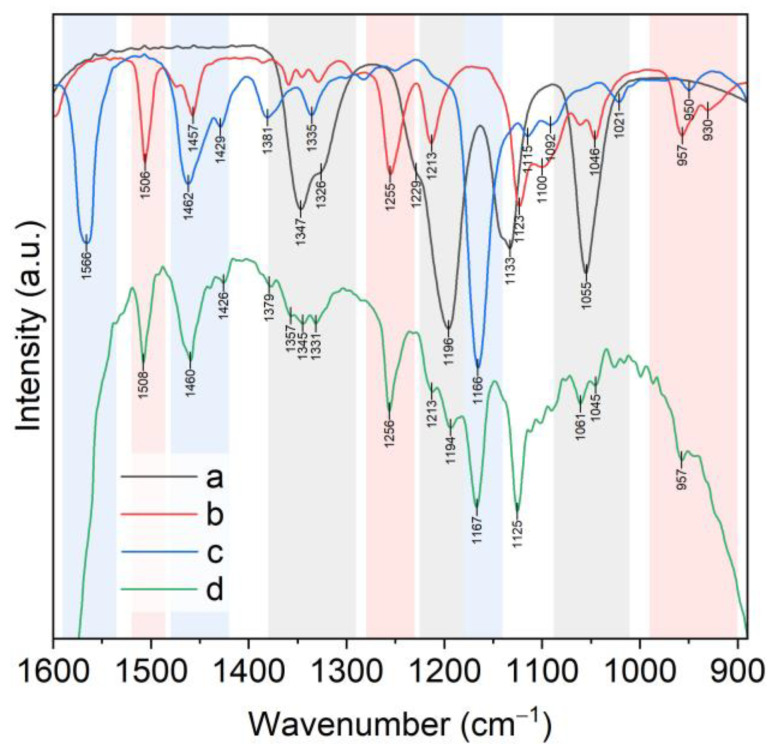
FTIR spectra of aqueous solutions (a) LiNTf_2_; (b) [Li(B15C5)(H_2_O)Cl]; (c) ionic liquid [C8mim]Cl; (d) equilibrium aqueous phase of the extraction system LiCl-B15C5-[C8mim][NTf_2_].

Multiple conclusions can be made from the analysis of the aqueous phases. In the FTIR spectra of water phases of LiCl-B15C5-[C8mim][NTf_2_] (Figure 17), the bands corresponded to the [C8mim]^+^ (1571, 1467, 1169 cm^−1^), NTf_2_^−^ (1348, 1325 and 1189 cm^−1^), and B15C5 in the lithium complex (1508, 1459, 1255, 1123, 1050, 957 cm^−1^).

The ionic liquid [C8mim][NTf_2_] is practically insoluble in LiCl solution. These spectra show the absence of absorption bands characterizing [C8mim]^+^. Consequently, the ionic liquid constituents [C8mim]^+^ and NTf_2_^−^ found in the aqueous phase in the system with LiCl transferred to the aqueous solution as part of other compounds. We suggest that the transfer of these components in the aqueous phase proceeds the composition of [C8mim]Cl and [Li(B15C5)(H_2_O)_x_(NTf_2_)]. Combining these results with the results of ICP-MS determination of Cl^−^ anions in the extract, we can conclude that in the system with LiCl, the anhydrous [Li(B15C5)(NTf_2_)] complex is predominantly extracted.

### 2.6. Equations of the Extraction Reaction of LiCl and LiNTf_2_ with B15C5–[C8mim][NTf_2_]

Based on all of the information above, the following lithium extraction scheme by B15C5-[C8mim][NTf_2_] from LiNTf_2_ (1) can be proposed.

Li^+^_aq._ + NTf_2_^−^_aq._ + B15C5_org._ + H_2_O_aq._ ↔ [Li(B15C5)(H_2_O)(NTf_2_)]_org._ (1)

Spectrophotometric data for the system with LiNTf_2_ indicated a partial transition of B15C5 into the aqueous phase; therefore, it is highly possible that the resulting lithium complex partially participates in the interphase equilibrium (2).

[Li(B15C5)(H_2_O)(NTf_2_)]_org._ ↔ [Li(B15C5)(H_2_O)(NTf_2_)]_aq._ (2)

In the case of extraction from LiCl solutions, according to the results of infrared spectroscopic studies, an anhydrous lithium complex is formed in the organic phases. The ratios of the equilibrium concentrations of chloride ions and lithium in the organic phases suggest that the lithium complex with NTf_2_^−^ anion (3) is formed preferentially.

Li^+^_aq._ + Cl^−^_aq._ + [C8mim][NTf_2_]_org._ + B15C5_org._ ↔ [Li(B15C5)(NTf_2_)]_org._ + [C8mim]Cl_org._ (3)

The [C8mim]Cl salt formed as a result of ionic exchange participate in interphase equilibrium (4), with the main part of this salt being transferred into the aqueous phase.

[C8mim][Cl]_org._ ↔ C8mim^+^_aq._ + Cl^−^_aq._ (4)

Spectrophotometric data for LiCl systems indicated B15C5 transition to the aqueous phase. According to the IR spectroscopy, the B15C5 is present in the aqueous phase as a lithium complex (5). The presence of characteristic bands of NTf_2_^−^ anions and C8mim^+^ cations in the IR spectra of aqueous phases indicated the presence of these ions in the raffinate. It is known that [C8mim][NTf_2_] is practically insoluble in water and aqueous solutions of LiCl. Thus, it can be assumed that one or both of these components are included in the composition of water-soluble compounds. Moreover, with a high degree of probability, the NTf_2_^−^ is in a bound form, namely in a complex compound with lithium and B15C5. The presence of water in this complex compound cannot be determined.

[Li(B15C5)(NTf_2_)]_org._ + xH_2_O_aq_ ↔ [Li(B15C5)(H_2_O)_x_(NTf_2_)]_aq_ (5)

## 3. Materials and Methods

### 3.1. Extraction

The water phases are the salt solutions of LiCl or LiNTf_2_ in a wide range of concentrations. The organic phases are B15C5 (0.5 mol/L) solution in [C8mim][NTf_2_] or CHCl_3_. The phase volume ratio (O/A) in all cases was 1:1. The phases were mixed using the Biosan Multi-rotator Bio RS-24 at 25 °C for 30 min. Extraction systems were centrifuged using the Hettich EBA-200 at 6000 rpm for 5 min for phase separation. The concentrations of lithium and chloride ions in aqueous and organic phases were determined using inductively coupled plasma mass spectrometry (ICP-MS) (Agilent 7500ce). 

### 3.2. UV-Vis Spectroscopy

Transition of B15C5 in aqueous phase during extraction process was determined by UV-Vis spectroscopy based on the characteristic peak of benzene ring at 274 nm wavelength with the help of Jasco V-730. 

### 3.3. Synthesis 

Three complexes were synthesized: *[Li(B15C5)(H_2_O)(NTf_2_)] **(I)**, [Li(B15C5)_1.5_(H_2_O)](NTf_2_) **(II)***, and *[Li(B15C5)_2_(H_2_O)](NTf_2_) **(III)**.* A total of 1 g B15C5 was mixed with weighted portions of anhydrous LiNTf2 salt (1.07 g; 0.71 g; 0.53 g) corresponding to different molar ratios (1, 1.5, 2), then the mixture was dissolved in 50 mL of boiling CHCl_3_. The resulting solution was slowly evaporated in open air until a crystalline mass was formed. This crystalline mass was separated from the mother liquor via filtration. After air drying, the mass of formed crystals was determined, the yield was calculated, and elemental analysis was performed. Elemental analysis was performed on a C, H, N, S-analyzer «EUROVECTOR EURO EA 3000» (Carlo Erba Strumentazione). The results of the synthesis are presented in Table S1. 

### 3.4. X-ray Diffraction

A single crystal of each compound was mounted at a D8 Venture diffractometer under a stream of cooled nitrogen, where the crystallographic parameters and intensities of X-ray reflections were measured (graphite monochromatized λMoK_α_ radiation, λ = 0.71073 Å, ω-scan mode). A reduction of the experimental reflections was performed using the SAINT program [35]. The SADABS [36] program was applied for absorption correction. 

Structures (***I***) and (***II_mon_)/(II_tr_***) were solved via direct methods and refined via least squares against *F*^2^ in anisotropic approximation for non-hydrogen atoms. The positions of hydrogen atoms were calculated and refined using the riding model. In structure (***II_tr_***), rather high picks of residual electronic density (~3 e/Å3) were found. These were located in the vicinity of the first and fourth anions and highlighted a low-accuracy location, at least in these fragments of the structure. Most likely, this is associated with somewhat of a disorder of these anions due to disregarding minor components of the disorder. Taking into account the NTf_2_^−^ flexibility, one may suppose an occurrence of several such components.

This explains a complex view of the residual electron density, which represents the superpositions of many components of the disorder. In spite of an inevitable decrease in the accuracy of the study, the hydrogen atoms of the coordinated water molecules are located objectively in the residual Fourier synthesis. In addition, the cation structures were determined with little error. The structure of (***II_mon_***) was determined with high accuracy. 

In structure of (***III***) on reflection indexing, two variants of the space group were determined. One of them was monoclinic C, with the parameters a = 28.835 Å, b = 12.018 Å, c = 22.142 Å, α = 90°, β = 100.89°, γ = 90°, and V = 7535.2 Å^3^ (Rsym= 0.149). The second variant was a triclinic unit cell, whose parameters were a = 12.018 Å, b = 15.620 Å, c = 22.142 Å, α = 79.953°, β = 90°, γ = 67.374°, and V = 3767.5 Å^3^ (Rsym = 0). Possible space groups included *C*2/*m* (CFOM = 2.16), C2 (CFOM = 3.73) and P $\overset{-}{1}$. An attempt to solve the structure in the monoclinic space groups failed, whereas in the triclinic group, the structure was solved by direct methods and refined to R ~ 4 %. 

We failed to transit from the triclinic space group to a higher monoclinic one. Two anions independent to the triclinic group should have very close geometry, since in the monoclinic space group these species should be reduced to one. However, these anions show various corresponding torsion angles which do not coincide with the higher monoclinic space group.

In structure (***III***), we located two independent one-charged anions and four crown ether fragments in a cation, with electron density peaks in each cavity. This means that Li cations have partial occupations of their positions. The site occupation position (s.o.p.) of Li atoms was accepted to be 0.5. Final least square refinement with these values resulted in sensible values of thermal parameters for all atoms, with their values being close to each other.

All four structures were refined by least squares against F^2^ in the full-matrix anisotropic approximation for non-hydrogen atoms. Positions of hydrogen atoms were calculated (except for H atoms of water molecules in (***II***)) and refined using the riding model. In structure (***III***), the hydrogen atoms of three water molecules were not determined. 

All the calculations were carried out using the software SHELXTL-Plus and Olex-2 [37,38]. 

The main characterizations of X-ray experiments and parameters of structure solution and refinement are listed in Table 3.

CCDC No.s 2220399 (***I***), 2220403 (***II_mon_***), 2220402 (***II_tr_***), and 2227022 (***III***) contain the supplementary crystallographic data for this paper. These data can be obtained free of charge from The Cambridge Crystallographic Data Center via http://www.ccdc.cam.ac.uk (accessed on 1 November 2022).

### 3.5. ATR FT–IR Spectra

FT–IR spectra were measured using a JASCO FT/IR-6600 spectrometer on an ATR PRO ONE Technologies attachment with a diamond crystal PKS-D1F, using the ATR method in the range of 4000–250 cm^−1^. Solid samples were placed on the diamond crystal without sample preparation. When analyzing the organic phases (extracts), the preconcentration was performed by removing the solvent under vacuum.

## 4. Conclusions

Extraction of lithium from aqueous solutions of LiNTf_2_ and LiCl salts was studied when using benzo-15-crown 5 ether (B15C5) as an extractant in the ionic liquid [C8mim][NTf_2_]. It was shown that the system with LiNTf_2_ has significant advantages over the systems with LiCl (including better extraction, lower transfer of the extractant into the aqueous phase, and no loss of ionic liquid), provided that the concentration of LiNTf_2_ does not exceed 2 M.

LiNTf_2_ complexes with B15C5 with different LiNTf2:B15C5 ratios were first isolated and characterized by PCA and IR spectroscopy, viz: *[Li(B15C5)(H_2_O)(NTf_2_)] **(I)**, [Li(B15C5)_1.5_(H_2_O)](NTf_2_) **(II)**, [Li(B15C5)_2_(H_2_O)](NTf_2_) **(III)***.

Based on the extraction data and IR spectroscopic investigation of the extraction systems, it was found that the extraction of LiNTf_2_ by the B15C5-[C8mim][NTf_2_] system extracts the complex of composition (***I***)-*[Li(B15C5)(H_2_O)(NTf_2_)]*. When lithium is extracted from LiCl aqueous solution, an anhydrous complex composition-*[Li(B15C5)(NTf_2_)]* is primarily formed.

## Figures and Tables

**Table 1:** Vibration frequencies of ethylene glycol groups of B15C5 in complexes (***I–III***) and free ligand in FTIR spectra (cm^−1^).

Assignments	B15C5	(*I*)	(*II*)	(*III*)
ν_S_(COC)	981	957	963 954	965 947
ν(CO) + ν(CC) + ρ(CH_2_)	- 930 917 905	- 931 915 -	942 929 917 -	938 929br 912 -
ρ(CH_2_)	850	851	864 847 -	866 846 843
ρ(CH_2_) + ν(CO) + ν(CC)	822	836	833	833

**Table 2:** Vibration frequencies of coordinated water molecules in spectra (***I–III***) (cm^−1^).

Complex	ν(OH)	δ(H_2_O)
[Li(B15C5)(H_2_O)(NTf_2_)] (***I***)	3581, 3514	1632
[Li(B15C5)_1.5_(H_2_O)(NTf_2_)] (***II***)	3551, 3469	1647
[Li(B15C5)_2_(H_2_O)(NTf_2_)] (***III***)	3575, 3511, 3442	1633

**Table 3:** Crystallographic parameters, characterization of X-ray data collection, and structure refinement for (***I***), (***II_tr_***), (***II_mon_***), and (***III***).

Compound	(*I*)	(*II_tr_*)	(*II_mon_*)	(*III*)
Formula	C_16_H_22_F_6_LiNO_10_S_2_	C_46_H_64_F_12_Li_2_N_2_O_25_S_4_	C_46_H_64_F_12_Li_2_N_2_O_25_S_4_	C_60_H_78_F_12_Li_2_N2O31S4
System, sp. gr., Z	Monocl., P21/c, 4	Tricl., P $\overset{-}{1}$, 4	Monocl. *Pc*, 2	Tricl., *P* $\overset{-}{1}$, 2
*a*, Å	8.3734 (12)	12.1261 (11)	13.6943(14)	12.0183 (10)
*b*, Å	21.961 (3)	22.4203 (18)	14.3352(15)	15.6197 (13)
*c*, Å	12.968 (2)	24.106 (2)	16.8203(18)	22.1422 (18)
*α*, °	90	105.493 (2)	90	79.953 (2)
*β*, °	96.154 (5)	102.585 (3)	111.917(4)	90.00
*γ*, °	90	94.132 (3)	90	67.37
*V*, Å^3^	2371.0 (6)	6104.4 (9)	3063.4(6)	3767.5 (5)
*d*, g/cm^3^	1.606	1.540	1.534	1.493
Radiation; λ, Å	MoKα (λ = 0.71073)	MoKα (λ = 0.71073)	MoKα (λ = 0.71073)	MoKα (λ = 0.71073)
*μ*, cm^−1^	0.324	0.273	0.272	0.241
T, K	100	100	100	100
Sample size	0.42 × 0.38 × 0.36	0.24 × 0.14 × 0.04	0.22 × 0.18 × 0.16	0.16 × 0.14 × 0.1
Diffractometer	SMART APEX-II	VENTURA D8	VENTURA D8	VENTURA D8
Absorption correction; T_min_, T_max_	SADABS; 0.9207, 0.9705	SADABS; 0.8620, 0.9705	SADABS; 0.8215, 0.9705	SADABS; 0.8913, 0.9579
2θ_max_, deg.	54.2	54.0	54.4	54.7
Interval *h*, *k*, *l*	−10 ≤ h ≤ 10, −28 ≤ k ≤ 28, −16 ≤ l ≤ 16	−14 ≤ h ≤ 14, −27 ≤ k ≤ 27, −29 ≤ l ≤ 29	−17 ≤ h ≤ 17, −18 ≤ k ≤ 18, −21 ≤ l ≤ 21	−15 ≤ h ≤ 15, −20 ≤ k ≤ 20, −28 ≤ l ≤ 28
Reflections number: measured./uniq. (N_1_), R_int_ c I > 2σ(I) (N_2_)	40,042/5160, 0.0285, 4918	70,642/23,889, 0.0395, 19391	80,818/13,529, 0.0335, 13221	46,247/8969, 0.0438, 7686
Refinement	Least squares on F^2^	Least squares on F^2^	Least squares on F^2^	Least squares on F^2^
Variables	333	1647	824	1018
R_1_/wR_2_ on N_1_	0.0306/0.0775	0.1207/0.2777	0.0217/0.0553	0.0523/0.1266
R_1_/wR_2_ on N_2_	0.0294/0.0765	0.1053/0.2666	0.0208/0.0549	0.0420/0.1212
GOOF	1.022	1.041	1.015	1.012
Δρ_min_/Δρ_max_, e/Å^3^	−0.35/0.54	−1.48/3.27	−0.26/0.22	−0.28/0.40

## Data Availability

Not applicable.

## Supplementary Materials

The following supporting information can be downloaded at: https://www.mdpi.com/article/10.3390/molecules28030935/s1, Figure S1: FT-IR spectrum (a) B15C5; (b) [C8mim][NTf_2_]; (c) [Li(B15C5)(H_2_O)(NTf_2_)]; (d) [Li(B15C5)_1.5_(H_2_O)(NTf_2_)]; (e) [Li(B15C5)_2_(H_2_O)(NTf_2_)]; Figure S2: FTIR-spectra of LiNTf_2_ blank extraction in [C8mim][NTf_2_] with different [Li^+^] content; Figure S3: FTIR-spectra of LiCl blank extraction extract in [C8mim][NTf_2_] at different concentrations of [Li^+^] in equilibrium aqueous phase; Figure S4: FTIR-spectra of LiNTf_2_-B15C5 (0.5 mol/L)-[C8mim][NTf_2_] extract at the different [Li^+^]/[B15C5] ratio; Figure S5: FTIR-spectra of LiCl-B15C5 (0.5 mol/L)-[C8mim][NTf_2_] extracts at different [Li^+^]/[B15C5] ratio; Figure S6: FTIR-spectra (1) solution of the complex (I) in [C8mim][NTf_2_] (0,5 mol/L); (2) extract of LiNTf_2_-B15C5 (0.5 mol/L)-[C8mim][NTf_2_]; (3) extract of LiCl-B15C5 (0.5 mol/L)-[C8mim][NTf_2_]; Table S1: The results of the synthesis of complexes I–III.

## Sample Availability

Samples of the compounds are available from the authors.
