# Supplementary material for: Study of Lithium-Extraction Systems Based on Benzo-15-Crown-5 Ether and Alkylimidazolium-Based Ionic Liquid
Source: Molecules. 2023 Jan 17;28(3):935. doi: 10.3390/molecules28030935 (PMC9920876; doi:10.3390/molecules28030935)
Supplement: Supplementary file 1 [file molecules-28-00935-s001.zip › molecules-2103371-supplementary.pdf]

Appendix A. Supplementary data

«Study of lithium-extraction systems based on benzo-15-crown-5 ether and alkylimidazolium-based ionic liquid»

Alexey A. Bezdomnikov [1,*], Liudmila I. Demina [1], Lyudmila G. Kuz'mina [2], Galina V. Kostikova [1], Valeriy I. Zhilov [1] and Aslan Yu. Tsivadze [1,*]

[1] Frumkin Institute of Physical Chemistry and Electrochemistry of Russian Academy of Sciences,
Leninsky pr. 31-4, Moscow 119071, Russia; ionh1961@rambler.ru (L.I.D.); galyna_k@mail.ru (G.V.K.); zhilov@bk.ru (V.I.Z.)

[2] Kurnakov Institute of General and Inorganic Chemistry of Russian Academy of Sciences, Leninsky pr. 31, Moscow 119991, Russia; kuzmina@igic.ras.ru

* Correspondence: bezdomnikovaa@phyche.ac.ru (A.A.B.); tsiv@phyche.ac.ru (A.Y.T.)

Contents:

Figure S1. FT-IR spectrum a) B15C5; b) [C8mim][NTf$_2$]; c) [Li(B15C5)(H_2O)(NTf$_2$)]; d) [Li(B15C5)$_{1.5}$(H_2O)(NTf$_2$)]; e) [Li(B15C5)$_2$(H_2O)(NTf$_2$)]. 3

Figure S2. FTIR-spectra of LiNTf$_2$ blank extraction in [C8mim][NTf$_2$] with different [Li^+] content. 4

Figure S3. FTIR-spectra of LiCl blank extraction extract in [C8mim][NTf$_2$] at different concentrations of [Li^+] in equilibrium aqueous phase. 5

Figure S4. FTIR-spectra of LiNTf$_2$-B15C5 (0.5 mol/L)-[C8mim][NTf$_2$] extract at the different [Li^+]/[B15C5] ratio. 6

Figure S5. FTIR-spectra of LiCl-B15C5 (0.5 mol/L)-[C8mim][NTf$_2$] extracts at different [Li^+]/[B15C5] ratio. 7

Figure S6. FTIR-spectra 1) solution of the complex (***I***) in [C8mim][NTf$_2$] (0,5 mol/L); 2) extract of LiNTf$_2$-B15C5 (0.5 mol/L)-[C8mim][NTf$_2$]; 3) extract of LiCl-B15C5 (0.5 mol/L)-[C8mim][NTf$_2$] 8

Table S1. The results of the synthesis of complexes I – III. 9

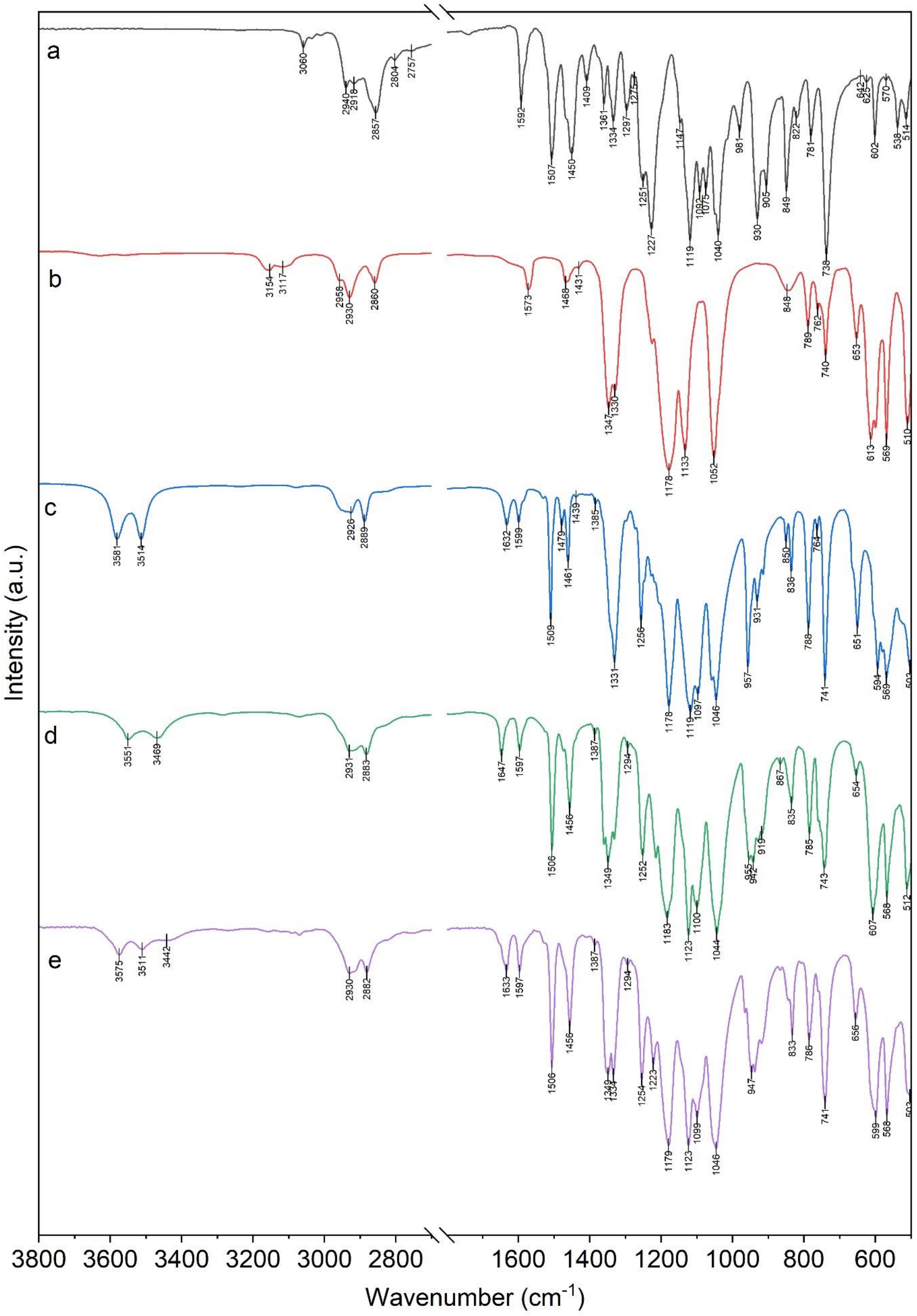

Figure S1. FT-IR spectrum a) B15C5; b) [C8mim][NTf_2]; c) [Li(B15C5)(H_2O)(NTf_2)]; d) [Li$(B15C5)_{1.5}$(H_2O)(NTf_2)]; e) [Li$(B15C5)_2$(H_2O)(NTf_2)].

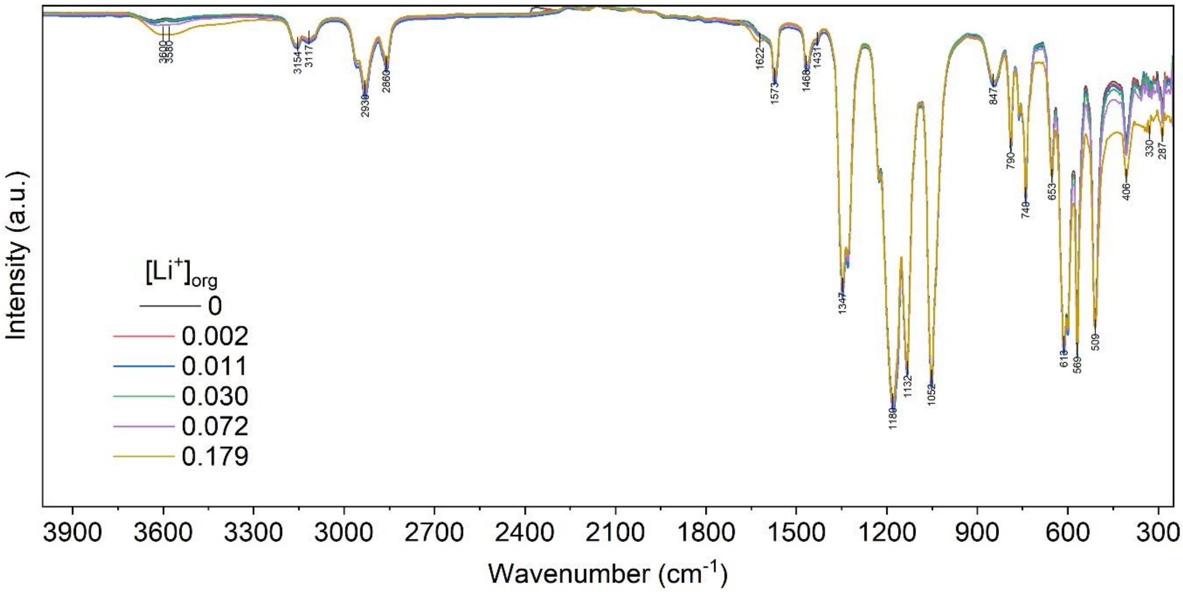

Figure S2. FTIR-spectra of $LiNTf_2$ blank extraction in [C8mim][NTf_2] with different [Li^+] content.

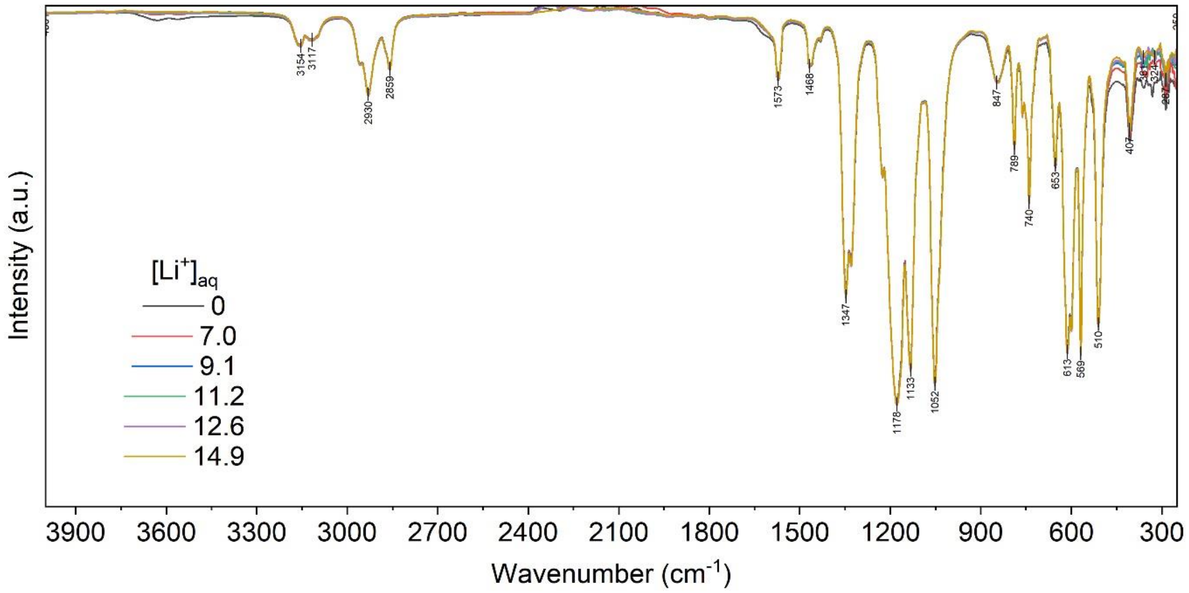

Figure S3. FTIR-spectra of LiCl blank extraction extract in [C8mim][NTf$_2$] at different concentrations of [Li$^+$] in equilibrium aqueous phase.

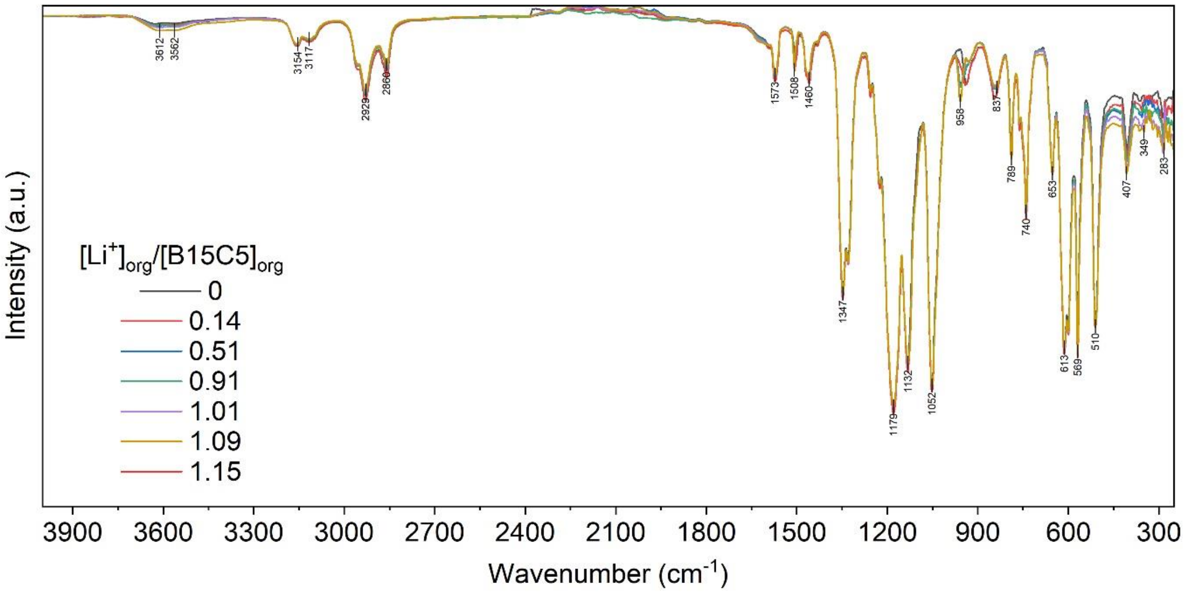

Figure S4. FTIR-spectra of $LiNTf_2$-B15C5 (0.5 mol/L)-[C8mim][NTf_2] extract at the different [Li^+]/[B15C5] ratio.

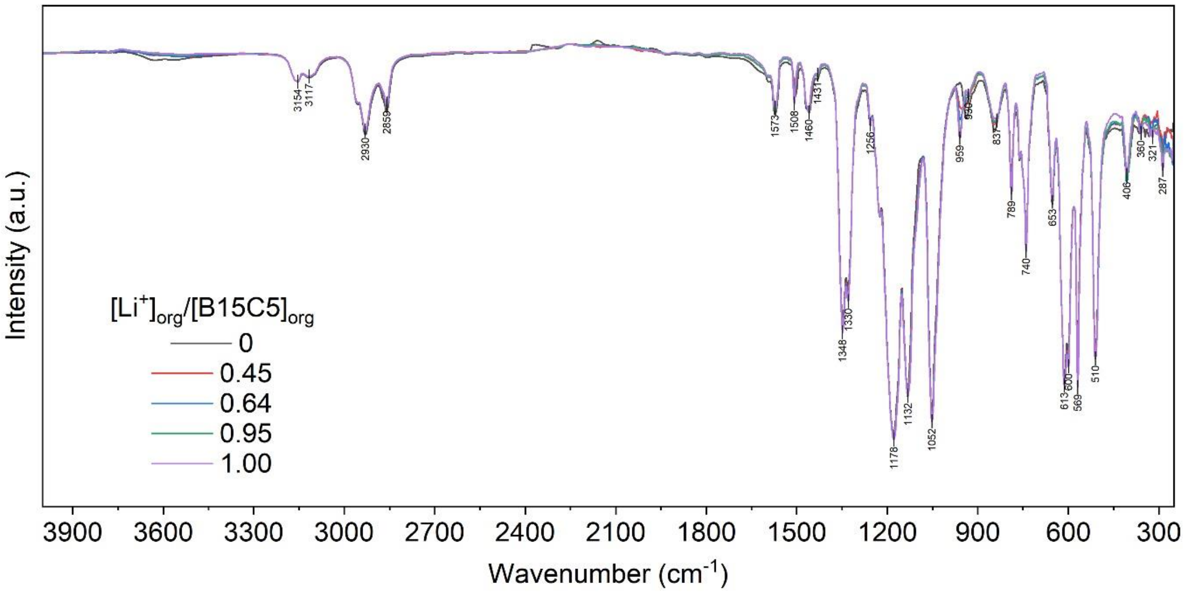

Figure S5. FTIR-spectra of LiCl-B15C5 (0.5 mol/L)-[C8mim][NTf_2] extracts at different [Li^+]/[B15C5] ratio.

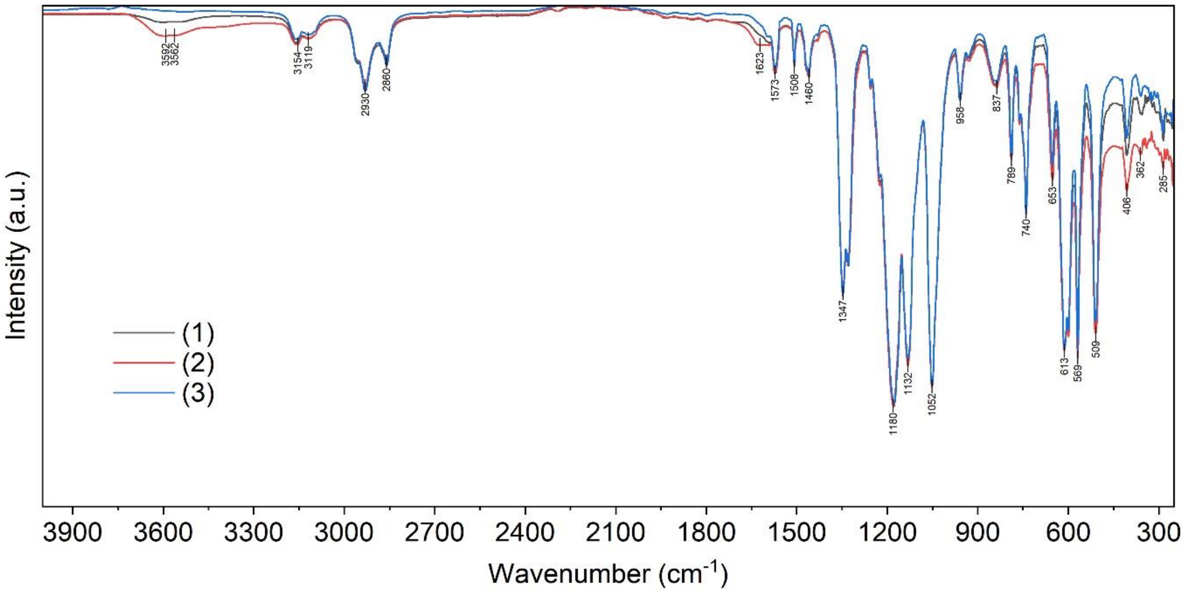

Figure S6. FTIR-spectra 1) solution of the complex (***I***) in [C8mim][NTf_2] (0,5 mol/L); 2) extract of $LiNTf_2$-B15C5 (0.5 mol/L)-[C8mim][NTf_2]; 3) extract of LiCl-B15C5 (0.5 mol/L)-[C8mim][NTf_2]

Table S1. The results of the synthesis of complexes I – III.

Compound	Yield, %	The results of elemental analysis
[Li(B15C5)(H_2O)(NTf_2)] (***I***)	76.84	N. 2.53; C. 34.70; H. 3.62; S. 11.60 (Calc.: N. 2.52; C. 34.60; H. 3.63; S. 11.55)
[Li(B15C5)$_{1.5}$(H_2O)](NTf_2) (***II***)	47.30	N. 2.02; C. 39.91; H. 4.41; S. 9.32 (Calc.: N. 2.03; C. 40.06; H. 4.39; S. 9.30)
[Li(B15C5)$_2$(H_2O)](NTf_2) (***III***)	51.49	N. 1.69; C. 43.68; H. 4.91; S. 7.82 (Calc.: N. 1.70; C. 43.74; H. 4.89; S. 7.79)
